# Acquired cystic kidney disease in patients on maintenance hemodialysis, prevalence and associated factors: a cross-sectional study

**DOI:** 10.11604/pamj.2023.45.175.31773

**Published:** 2023-08-21

**Authors:** Denis Georges Teuwafeu, Aicha Dongmo, Sylviane Dongmo Fomekong, Mballa Amougou, Maimouna Mahamat, Aristide Nono, François Folefack Kaze, Gloria Ashuntantang

**Affiliations:** 1 Department of Internal Medicine and Paediatrics, Faculty of Health Sciences, University of Buea, Buea, Cameroon,; 2 Faculty of Health Sciences, University of Buea, Buea, Cameroon,; 3 Department of Clinical Sciences, Faculty of Medicine and Pharmaceutics Sciences, University of Douala, Douala, Cameroon,; 4 Department of Nephrology, Faculty of Medicine and Biomedical Sciences, University of Yaoundé I, Yaoundé, Cameroon,; 5 Department of Nephrology, Yaoundé General Hospital, Yaoundé, Cameroon,

**Keywords:** Acquired cystic kidney disease, prevalence, associated factors, maintenance hemodialysis

## Abstract

**Introduction:**

Acquired Cystic Kidney Disease (ACKD) is a known complication in patients on maintenance hemodialysis, and it is associated with a high risk of malignant transformation. There is a paucity of data on ACKD in sub-Saharan Africa. Objectives: To determine the prevalence and factors associated with acquired cystic kidney disease in patients on maintenance hemodialysis.

**Methods:**

patients on maintenance hemodialysis were screened for ACKD. Patients with hereditary cystic kidney disease were excluded. Renal ultrasounds were performed by two radiologists. ACKD was defined as 3 or more bilateral renal cysts in a small or normal size kidney. Associated factors were determined using logistic regression. A p-value <0.05 was significant.

**Results:**

a total of 158 participants were enrolled and 61.4% (97) were male. Their mean (SD) age was 45.8 (14.9) years. The median dialysis vintage was 33.5 [10.7-63.2] months. The mean (SD) length of the kidneys was 85.1 (17.5) mm on the left and 81.2 (17.1) mm on the right. The prevalence of ACKD was 31.6% (n=50). Septated cysts (4), calcification of the wall of the cysts (2), irregular thick calcified wall (1), septated cysts with calcification (1) and hemorrhagic cyst (1) cysts were also observed. Dialysis vintage > 36 months (OR 7.1, 95% CI: 3.3 - 15.5) and male sex (OR 2.6, 95% CI: 1.2-5.6) were independently associated with ACKD.

**Conclusion:**

the prevalence of ACKD is high in a population of Cameroonians on maintenance. This result calls for the implementation of strategies to screen for the condition and its complications.

## Introduction

Acquired Cystic Kidney Disease (ACKD) is a condition characterized by the development of multiple bilateral cysts in the kidneys of individuals who have non-cystic kidney disease as the primary cause of Chronic Kidney Disease (CKD) [1]. The prevalence ranges from 8.2% to 92% is high when a computed tomography scan (CT scan) is used for cysts detection, in prospective studies and when the population studied has been on dialysis for a long or is elderly [2-13]. There is no current consensus on the diagnostic criteria of ACKD [14]. The required cystic changes compatible with the diagnosis vary from as little as 3 to more than 5 cysts in each kidney leading to considerable differences in the prevalence of the disease in various publications [12,15]. It is well known that ACKD develops as a consequence of sustained uremia. However, the exact pathophysiology behind its development is not well known. Most researchers hypothesize that, profound loss of renal tissues in end stage renal disease triggers mitogenic signals (azotemic products, altered concentration of sodium and potassium, Renin Angiotensin Aldosterone System (R-A-A-S), inflammation and local growth factors) promoting hypertrophy and hyperplasia of tubular renal cells. This hypertrophy and hyperplasia in addition to restricted tubular fluid contribute to cysts formation [1,16]. Acquired Cystic kidney disease (ACKD) is usually discovered incidentally in the course of abdominal imaging procedures. Complications such as renal cell carcinoma and haemorrhage occur in 20% and 50% of patients with cysts respectively and symptoms such as flank pain and gross hematuria may occur [1] Gender and primary cause of renal disease have been found to be associated with development of ACKD. Improve access to hemodialysis in Cameroon during the last decade has led to increased survival on maintenance haemodialysis thus; increase the likelihood of complications such as ACKD with subsequent increase risk of malignant transformation [17-19]. However, screening for ACKD and its complications is not routine. The aim of this study was to measure the prevalence of ACKD in patients on maintenance haemodialysis in Yaoundé, to report the ultrasonography findings of the complications of ACKD and to identify factors associated with ACKD.

## Methods

**Study design, setting, and sampling:** we conducted a hospital-based cross-sectional study, with data collected over a period of four months (January to April 2020), in the hemodialysis units of Yaoundé General Hospital (YGH) and Yaoundé University Teaching Hospital (CHUY). Both centers are government-funded hospitals, and offer two dialysis sessions of 4 hours twice a week. Only the dialysis sessions are subsidized. The patients pay out of pocket for any other prescriptions: the creation of vascular access, drugs, and laboratory test. Abdominal ultrasound is not routinely done. Cochran´s formula was used for sample size calculation the sample size and the minimal sample size was 153 participants. We included all patients with kidney failure on maintenance hemodialysis for at least 3 months. Patients with known cystic kidney disease as the primary cause of renal failure and patients with bilateral nephrectomy were excluded. Participants were seen at their dialysis center and on their dialysis days. The sampling method was consecutive and non-probabilistic.

**Data collection:** the presence of renal cysts was observed through ultrasounds performed by two radiologists using NEMIO MX® Toshiba, Japan Care, 2011 and ARIETTA V70® Hitachi Aloka, Healthcare Americas 2016 ultrasound machines. Cysts were visualized in the subcostal, ventral and or dorsal positions. Socio-demographic information such as age and sex, and dialysis-related information were recorded.

**Definitions:** acquired cystic kidney disease (ACKD) was defined as the presence of 3 or more bilateral cysts in small or normal-sized kidneys and, in children, as kidney cysts occurring de novo in a small or normal size kidney. The simple renal cyst was defined as less than 3 kidney cysts bilaterally or unilateral kidney cysts. Complications were the presence of ACKD and complex cysts, hemorrhagic cysts, abscess formation or kidney stones, and renal mass.

**Data analysis:** categorical variables are expressed as frequencies and percentages, while quantitative variables are expressed as mean and standard deviation for normally distributed variables and median and 25^th^-75^th^ percentiles for skewed variables. The chi-square and Fischer exact test were used to compare categorical variables as appropriate in the univariate analysis. Variables statistically significant in the univariate model were used for analysis in the multivariate analysis. All statistical analyses were performed using SPSS version 23 IBM and a p-value ≤0.05 was taken to indicate significance.

**Ethical consideration:** ethical approval (Ref: 2020/1102-01/UB/SG/IRB/FHS) was obtained from the institutional review board of the faculty of health sciences, of the University of Buea. The study conformed to the principles outlined in the Declaration of Helsinki. Written informed consent was obtained from each eligible participant before inclusion.

## Results

**Figure 1:**
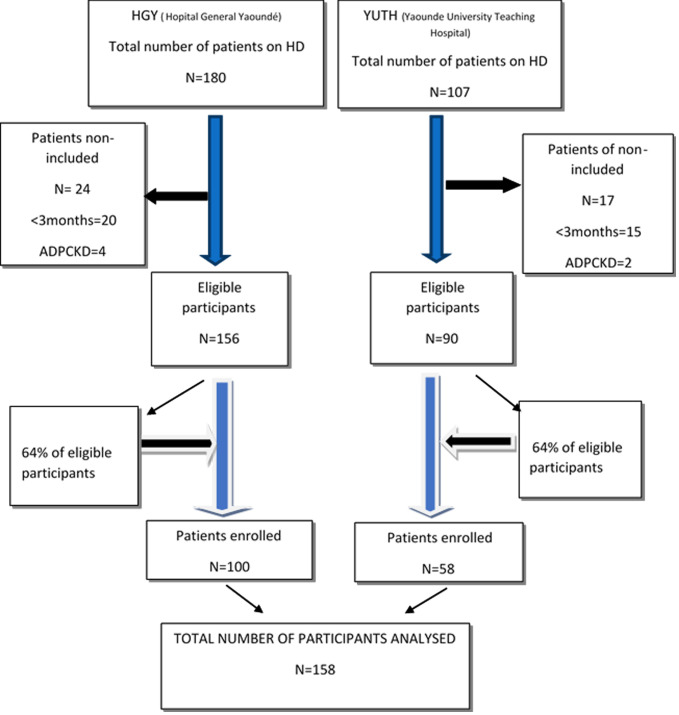
enrolment flowchart

Out of the 287 patients on maintenance hemodialysis in both hospitals, 246 met the inclusion criteria. We consecutively selected 64% of eligible participants in each hospital, giving a total of 158 participants (Figure 1).

**General characteristics of the study population:** we included 158 participants, 61.4% (n=97) were males. Their mean age (SD) was 45.8 (14.9) years, with 58.9% (n=93) of the population aged less than 50 years (Table 1). Half of the population had been on dialysis for less than 03 years. The median (25^th^-75^th^ percentile) dialysis vintage was 33.5 [10.7-63.2] months. Hypertension was the most prevalent comorbidity (n=136, 86.1%). Hypertension and chronic glomerulonephritis (CGN) were the main etiologies of End-stage kidney disease (ESKD). Anaemia was present in 86.1% (n=136) of the population, and 73.4% (n=116) of participants had no residual diuresis (Table 1).

**Table 1:** socio-demographic and clinical characteristics in the study population (N=158)

Variables	Proportion(n)	Percentage (%)
**Age ranges (years)**		
<50	93	58.9
≥50	65	41.1
**Gender**		
Male	97	61.4
Female	61	38.6
**Dialysis vintage ranges(months)**		
[3-37]	83	52.5
[37-122]	65	41.2
≥122	10	6.3
**Comorbidities**		
Hypertension	136	86.1
Heart failure	27	17.1
Diabetes mellitus	21	13.3
HIV infection	12	7.6
Macroscopic hematuria	8	5.1
Smoking	3	1.9
**Etiology of ESKD**		
Hypertension	54	34.2
CGN	36	22.8
Unknown	25	15.8
Chronic interstitial nephritis	15	9.5
Diabetes mellitus	12	7.6
Ischemic nephropathy	8	5.1
HIV	6	3.8
Cortical necrosis	2	1.3
**Complications of ESKD**		
Anemia	136	86.1
2° hyperparathyroidism	29	18.3
None	10	6.3
Adynamic bone disease	6	3.8
Anuria	116	73.4

HIV: human immunodeficiency virus. CGN: Chronic glomerulonephritis, ESKD: End-stage kidney disease

**Figure 2:**
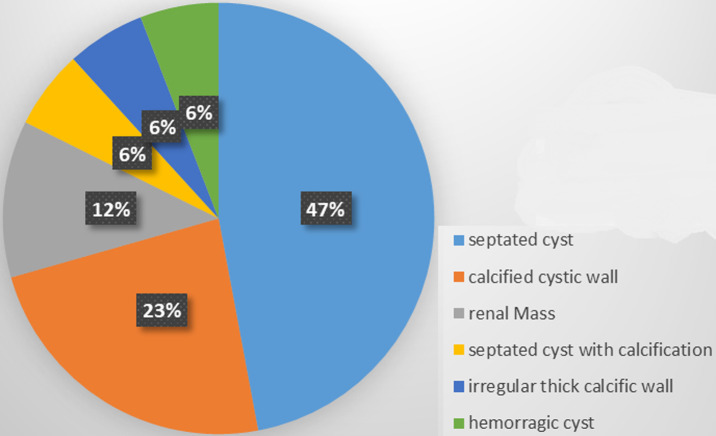
complicated cysts characteristics in participants with ACKD

**Prevalence and type of cysts:** the mean (SD) pole-to-pole length of the kidneys was 85.1(17.5) mm on the left and 81.2(17.2) mm on the right. Renal cysts were seen in 63.3 % (n=100) of participants, 43 % (n=69) were bilateral. The median [25^th^-75^th^ percentile] number of cysts in the right kidney was 4 (2-7) and 3 (2-7.2) in the left kidney (Table 2). The prevalence of ACKD was 31.6%. Out of the 50 participants with ACKD, 20 % (n=10) had complications, among which the most common were septated cysts (4) and calcified cysts (2). However, one patient had a mass (Figure 2).

**Table 2:** sonographic renal findings in the study population (N=158)

Variables	Number (n)	Percentage (%)
Patients with cysts	100	63.3
Bilateral cysts	69	43.0
Unilateral cyst	31	19.6
Median [25^th^-75^th^ percentile] number of cysts		
Right kidney: 4[2-7]		
Left kidney: 3[2-7.2]		
Mean (SD) pole-to-pole length of the kidneys (mm)		
Left 85.1 (17.5)		
Right 81.2 (17.2)		

**Associated factors:** dialysis vintage > 36 months (AOR: 5.4, 95% CI: 3.3 - 15.5) and male sex (AOR: 2.5, 95% CI: 1.2-5.6) were independently associated with ACKD (Table 3).

**Table 3:** factors associated with ACKD (multivariate analysis)

Variables	OR (95% CI)	AOR	95% CI	P-value
Male sex	2.6 (1.2 – 5.6)	2.5	1.1 - 5.6	0.033
Dialysis vintage>36	7.1(3.3 – 15.5)	5.4	2.3 – 12.8	<0.001
2° hyperparathyrodism	2.9 (1.3 – 6.6)	2.3	0.8 - 6.1	0.102
Anuria	3.7 (1.4 – 9.4)	1.1	0.3 - 2.8	0.857

OR: odds ratio, CI: confidence interval, AOR: adjusted odd ratio, ACKD: Acquired cystic kidney disease (ACKD)

## Discussion

In this study, we sought to determine the prevalence, and identify factors associated with ACKD in patients on maintenance hemodialysis in Yaoundé. We observed a 31.6% (n=50) prevalence of ACKD with a superimposed renal mass in one patient. Dialysis vintage>36 months (OR 5.4, p<0.01) and male sex (OR 2.5, p=0.03) were independently associated with ACKD. In this study, we observed a 31.6% prevalence of ACKD with a superimposed renal mass in one patient. The prevalence of ACKD varies from 8.2%-92% depending on the sensitivity of the diagnostic technique, the characteristics of the population studied, the study design, and the definition used to establish the diagnosis. It is higher when CT scan, or pathological analysis are used as screening methods, in prospective studies, or when the population studied has been on hemodialysis for long [2-13]. A similar study conducted in Korea with similar design and population revealed the same prevalence (31%) [20]. However, this prevalence is higher than the 10% and 8.2% prevalence reported in cross-sectional studies conducted in the USA and in Iran respectively [2,4]. The difference could be explained by the fact that our population was on dialysis for a longer period of time (median duration on hemodialysis [25^th^-75^th^percentile] of 33.5 [10.7-63.2] months) compared to the aforementioned studies were 75% of patients were on maintenance hemodialysis for less than 1 year and the mean dialysis vintage was of 27 months respectively. Moreover, our case definition of at least three cysts bilaterally different Vs. at least five cysts bilaterally in the aforementioned studies may have contributed to our high prevalence. Some cross-sectional studies have reported a prevalence of 59% and 80 % in the USA [9] and Brasília [21] respectively, but they recruited only patients who have been on dialysis for at least 05 years and used of CT scan as a screening method. It is well known that ultrasound (used as a screening modality in our study) might be insensitive for the detection of cysts less than 2 cm in a small sclerotic kidney [22].

Renal cell carcinoma is the most concerning complication of ACKD given the fact that, cysts serve as *nidus* for malignant transformation. We found one participant out of 50 with ACKD with renal tumor. The same observation was reported in Iran [2]. Higher prevalence (4.2%) of RCC in ACKD was reported in a retrospective study in USA where pathological report of nephrectomy specimens were analyzed [23]. Our low prevalence can be explained by the fact that small tumor (<0.5 cm) are generally more common in ACKD associated renal carcinoma, and they are generally below the minimum size detected by ultrasound [24]. Otherwise, pathologic analysis and/or CT scan are more suitable for ACKD associated RCC detection. International collaborative studies found that during an average follow-up of 2.5 years, 3% of patients with ACKD developed kidney cancer [22]. In agreement with previous studies [2-13,25], we found longer duration on dialysis and male sex to be associated with ACKD. This could be explained by the possibility of accentuated tubular renal cell hyperplasia by the effect of androgens leading to cysts formation and hence predisposing male to cysts development [10].

Similar to some reported studies, we did not find age to be associated with ACKD [1,6,8-10]. This is in contrast with a cross-sectional study reported in Korea where age greater than 60 was associated with ACKD [20]. The observed difference could be explained by the relatively young age in our population; 58.9 % (n=93) were aged below 50 years compared to the aforementioned study where more than half of their patients were aged above 50 years [26] as it has been shown that by the age of 50 years in a normal population 1/3 (25%) of persons would have 1 to 2 renal cysts.

**Study limitations:** the limitations of this study included but not limited to: 1- The screening modality that was ultrasound may has failed to visualize small cysts in atrophied sclerotic kidneys of participants, thus, our prevalence could have been underestimated. In addition, the fact that ultrasound is operator dependent may have an effect on our reported prevalence. 2- The estimation of 24h urine output could be inaccurate because participants could have wrongly estimated it in addition, due to the unavailability of laboratories data, we were limited in assessing association between laboratories parameters (especially hemoglobin) and ACKD.

## Conclusion

The prevalence of ACKD in a population of patients on maintenance hemodialysis in Yaoundé is high. We found one patient with superimpose renal mass as a complication of ACKD. Dialysis vintage>36 months and male sex were independent factors with ACKD. Regular renal ultrasound, should be included in the follow-up of patients who have been on maintenance hemodialysis, specifically male patients who have been on dialysis for three years or more. Furthermore, a study using a CT scan as a screening method should be performed in the other to determine the real prevalence of ACKD and renal cell carcinoma.

### What is known about this topic

- *The prevalence of ACKD is 7-22% in the pre-dialysis population, the prevalence is 44% in less than 3 years after beginning dialysis and 79% more than 3 years after beginning dialysis; the prevalence of ACKD is up to 90% after 10 years on dialysis;*
- *Age greater than 60 years and prolonged duration on dialysis have been found to be primary risk factors of renal cyst development;*
- *Gender and primary cause of renal disease have been found to be associated with development of ACKD*.

### What this study adds

- *Screening for ACKD and its complications is not routine in our setting, so the magnitude of the situation is not known in our context;*
- *Dialysis population in Cameroon is extremely young, and the dialysis vintage shorter compare to developed country, there is a need to evaluate and come out with possible other associated factors than what is known in the literature*.
